# Cutaneous adverse drug reactions induced by Oxcarbazepine

**DOI:** 10.1186/2045-7022-4-S3-O12

**Published:** 2014-07-18

**Authors:** Yi-Hsin Hsiao, Wen-Hung Chung

**Affiliations:** 1 Drug Hypersensitivity Clinical and Research Center, Chang Gung Memorial Hospitals, Taipei, Linkou, Taiwan

## Poster background

Oxcarbazepine (OXC) is a structural analog of carbamazepine (CBZ). OXC is considered a promising alternative medication for patients who cannot tolerate CBZ because of its equivalent clinical effiecacy and fewer cutaneous adverse drug reactions (cADRs) compared to CBZ. HLA-B*15:02 allele was shown to strongly associate with carbamazepine (CBZ)-induced Stevens-Johnson syndrome (SJS)/toxic epidermal necrolysis (TEN) in Chinese and Southeast Asian populations.However,the epidemiological data about OXC-induced cADRs in recent years is limited. This study aims to investigate the clinical characteristics of OXC-induced cADRs and their genetic associations with HLA-B*15:02.

## Method

We conducted a perspective study of patients with OXC-induced cADRs from Chang Gung Memorial Hospital Health System and Taiwan-SCAR consortium since 2006 to 2013. The diagnosis of OXC-cADRs were based on clinical features, exclusions of alternative causes, supported by ancillary investigations such as histological and laboratory findings. Clinical course, latent period, drug dosage, organ involvement, complications and the mortality were analyzed. We also examined the HLA-A and -B genotypes of all patients with OXC-induced cADRs comparing to OXC-tolerant controls.

## Results

The study included 31 patients with OXC-cADRs, including 12 SJS (no TEN case), 4 drug rashes with eosinophilia and systemic symptoms (DRESS ), 13 maculopapular exanthema (MPE), 2 bullous fixed drug eruption (BFDE) and 101 OXC-tolerant controls. Comparing to CBZ, the clinical severity of OXC-induced SJS and DRESS were less severe. All cases of OXC-SJS presented with limited skin detachment (<5%) without mortality, although there were typical mucosal involvements and typical histopathologicl findings of epidermal necrosis. All four cases of OXC-DRESS also presented with less severe liver injuries. Interestingly, similar to CBZ-SJS, the HLA study showed HLA-B 15:02 allele was significantly associated with patients with OXC-SJS (P< 0.01), but was not associated with other phenotypes. However, HLA-A*3101 was not significantly associated with all phenotypes of OXC-cADRs.

## Conclusion

Our findings suggest that HLA-B*15:02 allele is significantly associated with OXC- SJS in Han Chinese. However, the genetic association is phenotype-specific (only for OXC-SJS) and the strength is less than CBZ-SJS/TEN. Furthermore, in comparison to CBZ, OXZ-SJS presented with less clinical severity and better clinical outcomes, suggesting a decrease of antigenicity for induction of SJS than CBZ.

